# Forty-Five Years of Marburg Virus Research

**DOI:** 10.3390/v4101878

**Published:** 2012-10-01

**Authors:** Kristina Brauburger, Adam J. Hume, Elke Mühlberger, Judith Olejnik

**Affiliations:** Department of Microbiology, School of Medicine and National Emerging Infectious Diseases Laboratories Institute, Boston University, 72 East Concord Street, Boston, MA 02118, USA; Email: brauburk@bu.edu (K.B.); hume@bu.edu (A.J.H.); jolejnik@bu.edu (J.O.)

**Keywords:** Marburg virus, filoviruses, epidemiology, ecology, clinical manifestations, virus structure, replication cycle, pathogenesis, animal models, vaccine development

## Abstract

In 1967, the first reported filovirus hemorrhagic fever outbreak took place in Germany and the former Yugoslavia. The causative agent that was identified during this outbreak, Marburg virus, is one of the most deadly human pathogens. This article provides a comprehensive overview of our current knowledge about Marburg virus disease ranging from ecology to pathogenesis and molecular biology.

## 1. Epidemiology

Marburg virus (MARV) first appeared in August 1967, when laboratory workers in Marburg and Frankfurt, Germany and Belgrade, Yugoslavia (now Serbia) were infected with a previously unknown infectious agent. The 31 patients (25 primary, six secondary infections) developed severe disease that progressed to a fatal outcome in seven of the cases. An additional case showing symptoms of disease was diagnosed retrospectively (reviewed in [1]). The source of infection was traced back to African green monkeys (*Chlorocebus aethiops*) that had been imported from Uganda and were shipped to all three locations. The primary infections ironically occurred when the monkeys were necropsied for the purpose of obtaining kidney cells to culture poliomyelitis vaccine strains. In the remarkable period of less than three months the etiologic agent was isolated, characterized, and identified by the joint effort of scientists in Marburg and Hamburg [2] and was later confirmed by Kunz and colleagues [3] and Kissling and colleagues [4]. The pathogen was named Marburg virus after the city with the most cases and represented the first isolation of a filovirus. Erroneously, a study published in ‘The Lancet’ claiming that the mysterious disease was caused by rickettsia or chlamydia has frequently been cited as the first report on the causative agent of Marburg virus disease (MVD) [5]. 

It was not until 1976 that the now better-known member of the family, Ebola virus (EBOV), first emerged in Africa [6,7]. Shortly thereafter marburgviruses and ebolaviruses were classified together in a newly established family termed *Filoviridae,* so-named after their distinctive thread-like structure (*filum* being Latin for thread).

**Figure 1:**
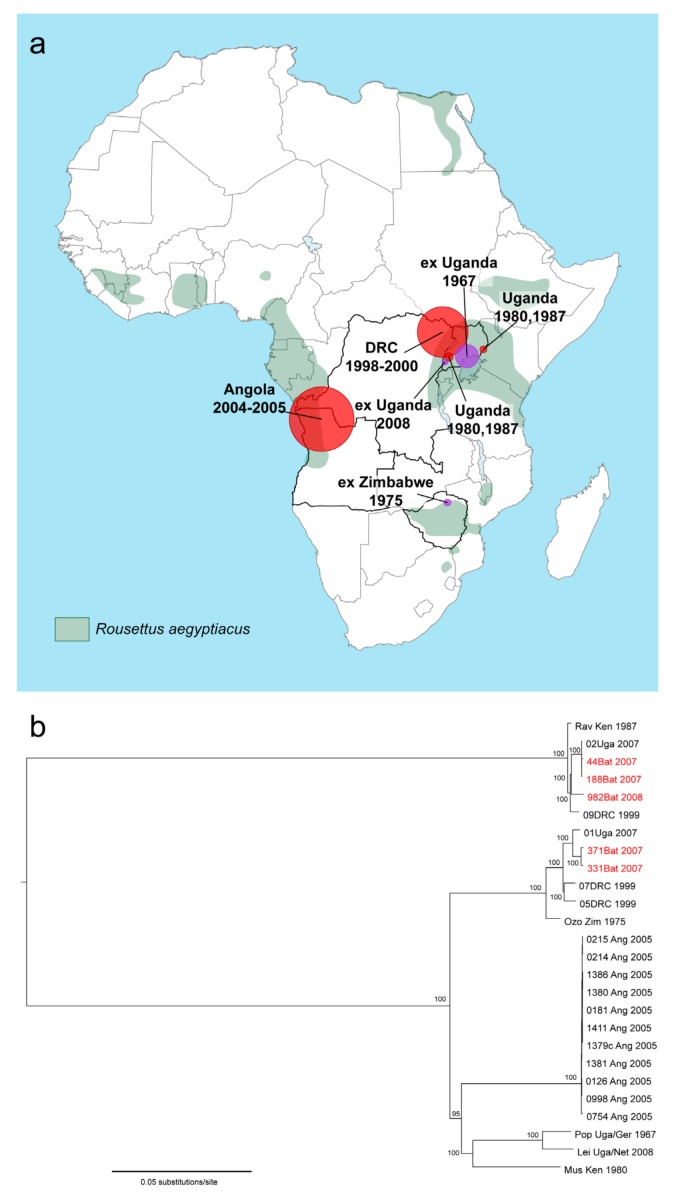
**Geographic distribution and phylogenetic analysis of Marburg virus. (a) **Location of Marburg virus (MARV) infections (circle sizes correspond to reported number of MARV cases) and distribution of the Egyptian fruit bat (*Rousettus aegyptiacus*) in Africa (www.iucnredlist.org). Outbreak locations (red circles) and sites of initial infection for exported cases of Marburg virus disease (MVD) (purple circles) are shown. **(b)** Bayesian phylogenetic analysis of full-length MARV genomes isolated from humans and bats. Numbers at the nodes represent posterior probability values. MARV isolates obtained from bats are shown in red. Analysis was performed by S. Carroll and J. Towner, Viral Special Pathogens Branch, CDC Atlanta, GA and represents an updated version of the analysis shown in [22].

MARV had not been heard of for eight years, when a young Australian who had traveled throughout Zimbabwe was admitted to a hospital in Johannesburg, South Africa with symptoms reminiscent of those observed during the 1967 outbreak in Europe [8]. When he died and the infection spread to his travel companion and later also to a nurse, Lassa fever was initially suspected resulting in strict barrier nursing techniques and isolation of the patients and their primary contacts. This lead to a quick containment of the outbreak, and while the secondary cases recovered, MARV was identified as the causative agent of the disease. In the following years from 1975 through 1985, only sporadic outbreaks that affected small numbers of individuals were caused by MARV on the African continent (Table 1, Figure 1a). As the case fatality rates associated with MVD were also lower than those seen in the devastating outbreaks associated with EBOV disease that reached up to 90%, MARV was long thought to be less threatening (Table 1). However, this view had to be revised as MARV reemerged in two large outbreaks occurring in the Democratic Republic of the Congo (DRC) in 1998–2000 [9] and then, for the first time also in Western Africa, in Angola, in 2004–2005 [10]. The total number of 406 cases and the high fatality rates (83% in DRC and 90% in Angola) revealed that MARV was as big of a threat for public health as EBOV [1,11]. The variation observed in disease severity and case fatality rates between these outbreaks *versus* the initial one in 1967 may depend on many complicating/mitigating factors. These include quality and availability of medical care, infectious dose and route of infection, differences in host population susceptibility (depending on immune and nutritional status) and genetics, inherent differences in viral variant virulence, and the prevalence of co-infections (particularly malaria and AIDS in patients from sub-Saharan Africa) [9]. The assumption that MARV Angola might be inherently more virulent than other MARV variants has been proposed mainly based on infection studies with nonhuman primates (NHP) [12,13,14] but is a matter of debate [15]. The genomes of the Angolan isolates differ about 7% at nucleotide level from the majority of the East African MARV isolates, including the ones from 1967 [10]. There is no evidence so far that the observed genetic differences result in higher virulence in humans. 

The DRC outbreak was unique, as there were at least nine different virus variants circulating in the tested patients indicative of several different spillover events from the natural reservoir to the human population [9]. In contrast, sequence data from the Angolan outbreak suggested a single introduction of MARV to an unidentified index patient and subsequent spread via person-to-person contact. The viral genomes showed a remarkably high genetic stability within this outbreak. Identical MARV genomes were isolated from patients even after two to three human to human transmissions [10].

To date there have been in total 452 cases and 368 documented deaths due to MVD. However, there is reason to assume that the actual numbers might be higher. During investigations of the 1998–2000 MVD outbreak in Durba, DRC, where primary infections were most likely acquired while working in a gold mine, it became apparent that there had been previous cases of so-called “hemorrhagic syndrome of Durba” that were associated with the mine but had been unreported since at least 1987. A nurse, who had survived one of these outbreaks in 1994, was later confirmed to have antibodies against MARV [9,16].

**Figure 2:**
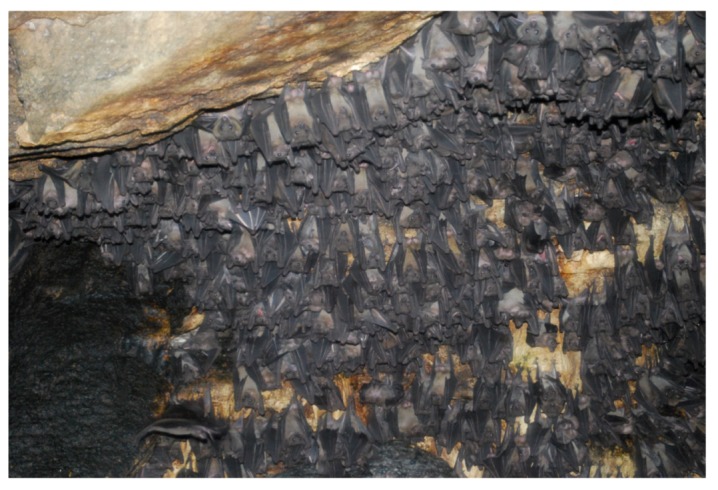
**Marburg virus reservoir.** Egyptian fruit bats (*Rousettus aegyptiacus*), the putative reservoir of MARV, roosting in the Python cave in Maramagambo Forest, Uganda. Two cases of MVD have been associated with visitors to this cave. Photo courtesy of Bobbie Rae Erickson, Viral Special Pathogens Branch, CDC, Atlanta.

Compared to an estimated 1.3 million deaths caused by HIV in 2009 in sub-Saharan Africa alone, MVD remains a rare disease in the endemic areas. Nevertheless, MARV also poses a risk for travelers to sub-Saharan Africa, and with their return home the chance increases for import and spread of MVD to other countries. This is underscored by the two most recent cases of MARV infections in 2008 of a Dutch and an American tourist who both presumably got infected during a visit to Python Cave in Uganda (Table 1, Figure 2). The Dutch woman succumbed to the infection after her return to the Netherlands, while the American tourist developed mild symptoms and survived [17,18]. In the nearby Kitaka mine in the Kamwenge district of Uganda, gold mining activity resulted in four cases of MARV infection in June–September 2007 [19]. Both locations were closed in response to the outbreaks.

In addition to these naturally occurring infections, two laboratory infections have been reported of which one had a fatal outcome [16,20].

Due to the lack of an approved vaccine or treatment, its high lethality and infectivity, and the potential of aerosol transmission, work with MARV is restricted to high-containment Biosafety Level 4 (BSL-4) laboratories [21]. Since MARV is considered to have the potential to pose a severe threat to public health and safety, it has also been classified as Select Agent by the Centers for Disease Control and Prevention (CDC), as Risk Group 4 agent by the World Health Organization (WHO) and as Category A Priority Pathogen by the National Institute of Allergy and Infectious Diseases (NIAID).

**Table 1:** **Marburg virus disease outbreaks. **History of all laboratory confirmed MVD cases. Table modified from [16].

Year(s)	City	Country	Origin (apparent or suspected)/ mode of infection	Fatalities/ number of cases (case fatality rate)	Isolate designation (abbreviation)	Reference
1967	Marburg	Germany	Uganda/	5/24 (21%)	MARV Ci67, Flak (MARV Flak), Hartz (MARV Hartz), MARV “L”, Porton (MARV Porton), Poppinga (MARV Pop), Ratayczak (MARV Rat), Voege (MARV Voe)	[23]
			Handling of infected African green monkey tissues			
1967	Frankfurt	Germany	Uganda/	2/6 (33%)		
			Handling of infected African green monkey tissues			
1967	Belgrade	Yugoslavia (now Serbia)	Uganda/	0/2 (0%)		
			Handling of infected African green monkey tissues			
1975	Johannes-burg	South Africa	Zimbabwe/	1/3 (33%)	Cruikshank (MARV Cru), Hogan (MARV Hogan), Ozolin (MARV Ozo)	[8,24]
			Unknown- most likely: sleeping in rooms inhabited by bats or visit to Sinoia caves (now Chinhoyi caves)			
1980	Nairobi	Kenya	Kenya/	1/2 (50%)	Musoke (MARV Mus)	[25]
			Working close to Kitum Cave, Mount Elgon National Park			
1987	Nairobi	Kenya	Kenya/	1/1 (100%)	Ravn (RAVV Ravn), R1 (RAVV R1)	[26]
			Visit to Kitum Cave/Mount Elgon National Park			
1988	Koltsovo	Soviet Union (now Russia)	Russia/	1/1 (100%)	“Variant U” (MARV “U”)	[16]
			Laboratory infection:Needlestick injury			
1990	Koltsovo	Soviet Union (now Russia)	Russia/	0/1 (0%)	-	[20]
			Laboratory infection:Unspecified violation of safety requirements			
1998-2000	Durba, Watsa (multiple indepen-dent, but simultan-eous or over-lapping outbreaks)	Democratic Republic of the Congo	Durba, Democratic Republic of the Congo/	128/154 (83%)	MARV 01DRC99, MARV 02DRC99, MARV 03DRC99, MARV 04DRC99, MARV 05DRC99, MARV 06DRC99, MARV 08DRC, MARV 10DRC99, MARV 11DRC99, MARV 12DRC00, MARV 13DRC00, MARV 14DRC00, MARV15 DRC00, MARV 16DRC00, MARV17 DRC00, MARV 18DRC00, MARV 19DRC00, MARV 20DRC00, MARV 21DRC00, MARV 22DRC00, MARV 22DRC00, MARV 23DRC00, MARV 24DRC00, MARV 25DRC00, MARV 26DRC00, MARV 27DRC00, MARV 28DRC00, MARV 29DRC00, MARV 30DRC00, MARV 31DRC00, MARV 32DRC00, MARV 33DRC00, MARV 34DRC00, MARV DRC 5/99 Aru, MARV DRC 5/99 Dra, RAVV 09DRC99	[9]
			Gold mining in Goroumbwa cave			
2004-2005	Uíge	Angola	Uíge Province, Angola/	227/252 (90%)	MARV Angola	[10,27]
			unknown			
2007	Kam-wenge	Uganda	Kamwenge District, Uganda/ Gold mining in Kitaka Cave	1/4 (25%)	MARV-01Uga 2007, RAVV- 02Uga 2007	[19]
2008	Colorado, City unreported	USA	Uganda/	0/1 (0%)	-	[17]
			Visit of Python Cave in Maramagambo Forest			
2008	Leiden	Nether-lands	Uganda/	1/1 (100%)	MARV Leiden	[18]
			Visit of Python Cave in Maramagambo Forest			
**Total:**				**368/452 (81%)**		

## 2. Ecology

MVD is considered a zoonotic disease that is thought to persist in a healthy reservoir host in the endemic areas in Africa. Humans and NHPs are spillover hosts and show a high rate of fatal disease outcomes. Several large-scale attempts to identify the natural host of filovirus infection throughout sub-Saharan Africa had been undertaken in the years since filoviruses first emerged with frustratingly little success [28,29,30,31]. Consistent with ecologic niche modeling of outbreaks and epidemiological patterns, isolated cases have suggested that EBOV is endemic in the rain forests of central and western Africa while MARV is more prevalent in open, dry areas of eastern, south-central Africa [32,33]. Almost all of the primary infections of natural MVD outbreaks so far have been linked to human entry into caves inhabited by bats (e.g., cave visitors, mine workers) (Table 1). Thus, bats have long been suspected to play an important role in the transmission cycle of the disease [31,32,34]. In 2007, evidence was detected for MARV infection of the common Egyptian fruit bat (*Rousettus aegyptiacus*) [35,36] (Figure 2), and MARV was isolated from healthy infected *R. aegyptiacus* bats caught in the same year in Uganda [22]. The bats were collected in Kitaka cave around the same time as human infections occurred that had been linked to the cave (Table 1 and see above, 1. Epidemiology). Genomic analysis of the few isolates of MARV acquired from bats showed that the sequences matched closely to the MARV genomes isolated from patient samples (Figure 1b). This was also the case for partial MARV sequences isolated from bats inhabiting the Goroumbwa mine in the DRC that was suspected to be the major location for several independent spillover events to gold miners between 1998 and 2000. The bat MARV sequences were closely related to the distinct isolates that had been reported during these outbreaks in humans [36].

A study analyzing MARV prevalence in bat populations in Gabon found MARV-specific nucleic acids in *R. aegyptiacus* bats in several local caves [37]. Together with previous data showing a high prevalence of MARV-specific antibodies in Gabonese bat populations [38] and with an observed relation of the isolated sequences with previously reported Gabonese bat isolates [35] this study suggests that MARV is enzootic in Gabon and raises the concern of further spread of MARV into other countries. Therefore, close ecological as well as serological surveillance of the bat populations in sub-Saharan Africa could help to predict and prevent further MVD outbreaks especially in areas where bats are still used as a food source.

It is not currently clear whether *R. aegyptiacus* bats are the exclusive reservoir for MARV or if other bat species reported to be positive for viral antibodies and RNA are also natural reservoirs or merely intermediate hosts [36].

## 3. Taxonomy

The genus *Marburgvirus* includes a single species, *Marburg marburgvirus* (formerly referred to as *Lake Victoria marburgvirus*) [39,40]. Phylogenetic analysis based on genomic sequence data suggests that the known members of this species can be assigned to at least five different lineages of which four are very closely related (nucleotide sequences differ up to 7%) while the fifth is divergent (a nucleotide difference of 21%) (Figure 1b) [10,22,39]. As the genomic divergence between all isolates is below 30%—the cutoff for the classification of the five different ebolaviruses into five different species—the five marburgvirus lineages were recently reclassified as two viruses. Ravn virus (RAVV) is represented by the Ravn isolates from 1987, one isolate from the DRC outbreak in 1998-2000, and one human and several bat isolates from infections that took place in Uganda in 2007. Marburg virus (MARV) is represented by all other sequenced isolates (Figure 1b, Table 1) [39]. For the sake of simplicity, in this review the abbreviation “MARV” is used for all marburgviruses and the abbreviation “EBOV” for all ebolaviruses.

## 4. Transmission

Initial MVD patients are believed to contract the virus via exposure to an infected animal: either a reservoir host (several bat species) or a spill-over host such as NHPs as described in the first MVD outbreak (see above, 1. Epidemiology) [23,41]. Following transmission to humans, spread of the virus between individuals is the result of direct contact with blood or other body fluids (saliva, sweat, stool, urine, tears, and breast milk) from infected patients. Typical risks of exposure include administration of medical care to infected individuals as well as handling of corpses without use of proper protection [34]. Of particular note, virus has been found in tears, semen, and in a liver biopsy weeks to months following the onset of symptoms highlighting the importance of monitoring convalescent patients [20,42,43,44].

## 5. Clinical manifestations

Much of what we know about typical MVD symptoms comes primarily from clinical data obtained during the three largest recorded MVD outbreaks: the 1967 outbreak in Germany and Yugoslavia, the 1998–2000 outbreak in the DRC, and the 2004–2005 outbreak in Angola. Although the case fatality rates were significantly higher in the latter outbreaks, most of the clinical symptoms observed were similar.

### 5.1. Incubation Period

Based on the most reliable documented cases of exposure and subsequent illness, MVD has an incubation period ranging from 3 to 21 days (typically 5 to 10 days), which is likely modulated by factors such as infectious dose and possibly by route of infection. The course of MVD has conventionally been broken down into three phases [45]: an initial generalization phase, an early organ phase, and either a late organ phase or convalescence phase depending upon disease outcome [45]. A summary of MVD symptoms is reviewed below [16,46,47,48,49].

### 5.2. Generalization Phase (Day One-to Four)

The onset of illness begins with generic flu-like symptoms; a characteristic high fever (typically 39–40 ^o^C), severe headache, chills, myalgia, prostration, and malaise. For many patients (50–75%) this is followed by rapid debilitation characterized by gastrointestinal symptoms including anorexia, abdominal pain, severe nausea, vomiting, and watery diarrhea. Starting on day four to five patients commonly develop enanthem, dysphasia, and pharyngitis. Additionally, a characteristic maculopapular rash is typically the first distinctive feature indicating a filovirus infection *versus* influenza or malaria. Other common symptoms include lymphadenopathy, leukopenia, and thrombocytopenia.

### 5.3. Early Organ Phase (Day Five to Thirteen)

Many of the initial symptoms may persist in the early organ phase, and patients may sustain a high fever. They may additionally display neurological symptoms including encephalitis, confusion, delirium, irritability, and aggression. Patients can also develop dyspnea and abnormal vascular permeability, particularly conjunctival injection and edema. During the latter part of this phase more than 75% of patients present with some form of clear hemorrhagic manifestation such as petechiae, mucosal bleeding, melena, bloody diarrhea, hematemesis, and ecchymoses. Due to the unusualness of hemorrhagic symptoms, diseases caused by filoviruses have sometimes been referred to as hemorrhagic fevers (Marburg Hemorrhagic Fever (MHF) and Ebola Hemorrhagic Fever (EHF)), although these terms are currently disfavored since not all patients display hemorrhagic symptoms. At this stage, multiple organs are affected including the pancreas, kidney, and liver. Elevated serum activity of a number of liver enzymes including SGOT and SGPT have been observed in most patients sampled.

### 5.4. Late Organ/Convalescence Phase (Day Thirteen+)

The late stages of MVD result in one of two potential outcomes: patients either succumb to the disease or enter a prolonged phase of recuperation. Typical preagonal symptoms include restlessness, obtundation, confusion, dementia, convulsions, reduced circulation due to severe dehydration, metabolic disturbances, severe diffuse coagulopathy, multiorgan failure, shock, and coma. Fatalities typically occur 8–16 days following the onset of symptoms, with death usually the resulting of shock and multiorgan failure. Non-fatal cases are typified by an extensive convalescent period during which myalgia, exhaustion, sweating, peeling of the skin at the sites of rash, partial amnesia, and secondary infections are all common.

## 6. Prevention

Prevention of newly emerging MARV infections and effective containment during ongoing outbreaks is both essential and challenging, as there is currently no licensed vaccine or treatment available for general use.

Following the 1967 outbreak of MVD in Europe and cases of infection with ebolavirus Reston in imported crab-eating macaques (*Macaca fascicularis*) in the USA in 1989/1990 and 1996 as well as 1992 in Italy (reviewed in [50]), strict quarantine procedures have been put in place that have so far prevented infections acquired by imported NHPs into non-endemic countries [51,52]. To avoid the spread of filoviruses by tourists, Python cave was closed to the public following the diagnosis of the Dutch patient in 2008.

The prevention and control of outbreaks and infections in endemic countries is much more challenging. In the past, joint efforts of teams from the WHO, Doctors Without Borders, the Red Cross, the CDC and others in collaboration with the local ministries of health have been undertaken to cease the spread of MVD. The main focus of outbreak control is the prevention of secondary transmission and further primary infections.

**Figure 3:**
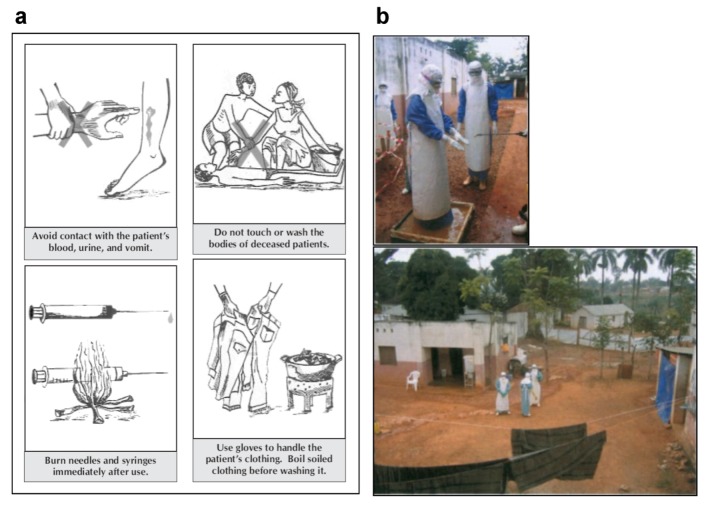
**Marburg virus disease outbreak control. (a)** Signs used to educate the local population in outbreak areas. Picture taken from [57]. **(b)** Pictures of the MVD outbreak in Angola, 2005. Above, nurse being sprayed with chlorine while leaving the isolation ward. This illustrates the protective clothing worn by nursing staff. Below, view showing a section of the isolation ward. The ward for confirmed MARV patients is on the left. The solid plastic sheeting used for the outer wall is shown in the distance. Figure and legend modified from [54].

The first measures in response to a MVD outbreak include setting up isolation wards in hospitals to assure rapid isolation of MARV-infected patients and prevent person-to-person transmission (Figure 3b). Proper and fast laboratory diagnosis of suspected cases is key to eliminate further spread. Nosocomial infections were commonly seen in earlier outbreaks [8,9,23]. However, reinforcement of barrier nursing techniques and education of health care workers have limited these infections in recent outbreaks (Figure 3a). Epidemiological surveillance has been crucial in the identification of index cases as well as the predominant modes of transmission. In endemic areas, secondary infections mainly occurred while taking care of ill patients and family members or during traditional burial practices involving close contact to corpses [53]. Therefore, the execution of safe burial and disinfection techniques and information campaigns to educate the local population are essential in order to contain the spread of infections in endemic areas (Figure 3a) [27,53,54].

Biosafety and epidemiological efforts alone were not sufficient for efficient outbreak control during large outbreaks, emphasizing the need for additional psychosocial support of the affected communities [53]. The fast progression and high lethality rates associated with MVD even—and especially—after hospital admission resulted in a high level of fear and suspicion by the resident population. The fact that health care workers wearing recommended personal protective equipment (PPE) were fully masked and not identifiable further increased anxiety (Figure 3b). This resulted in the hiding of infected family members and verbal and in some cases physical aggression towards members of aid organizations [53]. Communicating necessary protective measures while respectfully considering the affected families’ and communities’ traditions and culture during ongoing outbreaks is therefore essential for successful outbreak management.

The recent identification of bats as the potential reservoirs for MARV as well as EBOV [22,35,36,55,56] will help to increase not only the public awareness, but also the effectiveness of the preventive measures taken in endemic areas to minimize contact with infected animals (*i.e*. closing of bat inhabited caves for the public, serosurveillance of bat populations) [18,19]. This is a challenging task, emphasized by the fact that during the last cluster of MARV infections linked to a gold mine in Uganda, the miner hired to enforce the restricted access to the mine got infected. The mine had been closed in response to the ongoing outbreak and even though he was aware of the risk, he had entered the mine without the suggested PPE [19]. 

Later, the bat population of this mine was eliminated by the owner by means of fumigation [19]. As bats of most species are endangered, this does not seem a viable option and educational campaigns aimed at villagers living close to bat-inhabited caves as well as tourist groups and tour operators might prove more sustainable in the future.

## 7. Virus Structure

### 7.1. Virion Structure

**Figure 4:**
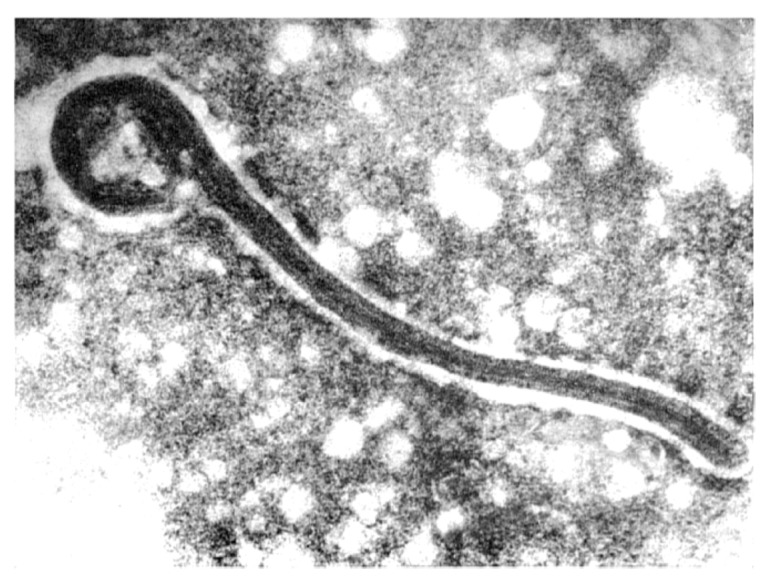
**The first electron micrograph of a Marburg virion from 1967.** Image courtesy of W. Slenczka, University of Marburg, Germany.

In 1967, during the first reported filovirus disease outbreak in Europe, the identification of the previously unknown causative agent of the deadly disease was performed by electron microscopy (EM) (Figure 4). The unusual filamentous structure of the particles led to some confusion and it was even suggested that the causative agent of the disease might be related to the spiral-shaped Leptospira, a genus of the spirochaetes bacteria [58]. Others concluded that the observed particles were viruses morphologically related to rhabdoviruses and named the newly discovered pathogen Marburg virus [23,59]. Marburg virions are pleomorphic particles, which appear as rod- or ring-like, crook- or six-shaped, or branched structures. Cryo-EM analysis of purified virions showed that about 30% of viral particles released from infected Vero cells were filamentous, 37% were six-shaped, and 33% were round [60]. The same study revealed a mean particle length of 892 nm and a mean diameter of 91 nm. Previous conventional EM studies showed that the MARV particles were uniformly 80 nm in diameter, whereas the length varied widely with virions measuring up to 14,000 nm. The average particle length was 790 nm [61,62,63]. The reported differences in particle size might be due to experimental differences between cryo-EM and conventional EM [60]. Notably, MARV particles are considerably shorter than EBOV virions, although MARV genomes are slightly longer than EBOV genomes [62,63].

**Figure 5:**
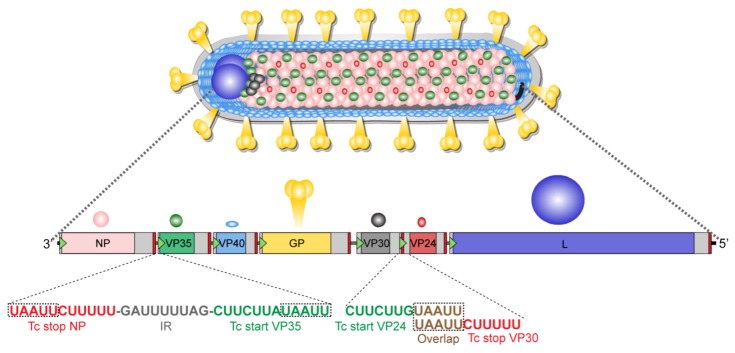
**Marburg virion structure and genome organization.** Above, schematic of Marburg virion. Below, structure of the MARV genome with transcription signals. The colors of the open reading frames correspond to the colors of the viral proteins. Untranslated regions of the different genes are shown as light grey boxes; intergenic regions (IR) are shown as dark grey lines and the leader and trailer of the genome are colored in black. Transcription start signals (Tc start) are represented by green triangles, while transcription stop signals (Tc stop) are shown as red bars. The sequence of two gene borders (NP/VP35 and VP30/VP24) is shown in 3’ to 5’ orientation, as it occurs in the negative sense RNA genome (MARV Musoke, GenBank accession number: NC_001608). The gene border between VP30 and VP24 contains overlapping transcription signals, with the start signal of VP24 upstream of the stop signal of VP30.

MARV particles are surrounded by a host-derived membrane that is coated with spikes of 5-10 nm in length, which are formed by trimers of the viral glycoprotein (GP) (Figure 5) [60,61,62,63,64,65]. The central core of the viral particles is the ribonucleoprotein complex (nucleocapsid) formed by the viral RNA genome and tightly associated nucleocapsid proteins (Figure 5). The nucleocapsids are highly organized tubular structures with an outer diameter of 45-50 nm and an electron-dense central axis of 19-25 nm. The central axis is surrounded by a helical capsid with cross-striations at a 5 nm interval [61,62,63]. A recently published detailed cryo-electron tomography analysis of MARV virions has shed some light on the structural organization of the nucleocapsids. Reconstructions of virion-associated nucleocapsids using subtomogram-averaging analysis revealed that the MARV nucleocapsids form a left-handed helix with a pitch of 7.5 nm and a flexible average symmetry of 14.96 protrusions per turn with two inner lobes of density per protrusion. The inner lobes represent the nucleoprotein (NP), suggesting that the MARV nucleocapsid contains an average number of 29.92 NP molecules per turn with each NP molecule packaging six RNA bases [60]. MARV nucleocapsids show directionality, having a pointed and a barbed tip [60].

### 7.2. Genome Organization

The nonsegmented negative-sense (NNS) RNA genomes of the various MARV isolates range in size from 19,111 to 19,114 nts and contain seven monocistronic genes in a linear order (Figure 5) [66,67]. Each gene is composed of a highly conserved transcription start and stop signal, an unusually long 3’ and 5’ untranslated region, and the open reading frame (ORF). The genes are either separated by short intergenic regions that range from 4 to 97 nts, or the transcription stop signal of the upstream gene and the transcription start signal of the downstream gene overlap, sharing five highly conserved nts (Figure 5). The structure of this gene overlap is found among all filoviruses and is unique among members of the order *Mononegavirales *(for review see [68]). The 3’ and 5’ genome ends are extracistronic regulatory regions that contain *cis*-acting signals essential for transcription and replication, including transcription and replication promoters.

There are generally two types of genomic replication promoters for NNS RNA viruses: a bipartite promoter found in members of the paramyxovirus subfamily *Paramyxovirinae* and one continuous more compact replication promoter for rhabdo- and pneumoviruses [69]. The bipartite promoter structure of the *Paramyxovirinae* subfamily is associated with the “rule of six”, *i.e*., the total genome length must be a multiple of six [70]. Given that filoviruses do not obey the rule of six, it was surprising that mapping of the MARV genomic replication promoter revealed a bipartite structure. The 3’ genome end, the leader, comprises 48 nts and contains the first promoter element of the bipartite genomic replication promoter. The second promoter element consists of a (UNNNNN)_3_ motif with three conserved uridine residues separated from each other by five non-conserved nucleotides. The UN_(5)_ hexamers are located within the 3’ untranslated region of the first MARV gene, the NP gene, and are separated from the first promoter region by the 12 nts long transcription start signal. Substitutions in the NP transcription start signal do not affect replication activity but do interfere with transcription initiation [71]. The 5’ extracistronic region, the trailer, spans the last 75 nts of the MARV genome and contains the complement of the antigenomic replication promoter (see below, 8.2. Transcription and Replication). The structure of the MARV antigenomic promoter has not yet been determined. However, due to the presence of UN_(__5)_ hexamers it is likely that it is of bipartite nature, similar to the genomic promoter. A common feature of the leader and trailer regions of all NNS RNA viruses is a high degree of complementarity of the 10-15 most terminal 3’ and 5’ nucleotides [72]. Although filoviruses share this feature, both the leader and the trailer also have the capability to form an internal secondary structure, which is not the case for the leaders and trailers of other NNS RNA viruses [71,73,74,75].

### 7.3. Viral Proteins

The MARV genome encodes seven structural proteins listed in Table 2. 

**Table 2:** **MARV proteins and their function.** The apparent molecular mass refers to the migration pattern of the proteins in SDS polyacrylamide gel electrophoresis and in some cases is different to the predicted molecular mass.

Protein	Amino Acids	Apparent Molecular Mass	Function
NP	695	94 kDa	encapsidation of RNA genome, nucleocapsid formation, budding, essential for transcription and replication
VP35	329	32 kDa	polymerase cofactor, nucleocapsid formation, IFN antagonist
VP40	303	38 kDa	budding, antagonist of IFN signaling
GP	681	170-200 kDa	attachment, receptor binding, fusion, tetherin antagonist
VP30	281	28 kDa	nucleocapsid formation
VP24	253	24 kDa	maturation of nucleocapsids, budding
L	2331	~220 kDa	catalytic domain of RNA-dependent RNA polymerase

#### 7.3.1. Glycoprotein (GP)

MARV has a single surface protein, GP, which is encoded by the fourth gene and mediates attachment to target cells and virus entry [76]. GP is a Type I transmembrane protein which is inserted into the viral envelope in the form of homotrimeric spikes [65]. In contrast to ebolaviruses, which use transcriptional editing to express the membrane-bound GP and at least two nonstructural glycoproteins [77,78,79,80], the MARV GP gene contains a single open reading frame (ORF) encoding the full-length GP. During its transport from the endoplasmic reticulum (ER) to the plasma membrane via the secretory pathway, the precursor GP is the target of various posttranslational modifications including glycosylation [65,81], acylation [82], and phosphorylation [83]. GP is heavily glycosylated by complex and high mannose-type *N*-linked glycans as well as by mucin-type *O*-linked glycans, with the carbohydrates contributing about 50% of the apparent molecular weight of the protein [65,84,85]. Similar to EBOV GP, the *O*-linked glycans and many of the *N*-linked oligosaccharides are clustered in a mucin-like domain [76]. After synthesis in the ER, the precursor GP is cleaved at amino acid 435 by furin or a furin-like protease in the *trans* Golgi network, resulting in two disulfide-linked subunits, GP_1_ (160 kD) and GP_2_ (38 kD) [86]. While the ectodomain, which is mainly formed by GP_1_, mediates binding to entry factors and receptors [87,88,89,90,91,92,93,94,95], the transmembrane subunit GP_2_ contains the fusion peptide and is presumed to mediate fusion of the viral and the cellular membrane based on similarity to EBOV GP_2_ both at the amino acid and structural level [96,97]. The 30 amino acid long transmembrane domain of GP_2_ is required for the incorporation of GP into virions [98]. In addition, the cytoplasmic tail of GP_2_ is involved in enhancing the efficiency of viral entry by maintaining the structure of the ectodomain [99]. The receptor binding domain of MARV GP was mapped to the aminoterminal region of GP_1_ spanning amino acids 38 to 188 [100], whereas the highly glycosylated mucin-like domain is not essential for virus entry [101]. An important step in MARV entry is the proteolytic activation of GP_1_ by endosomal proteases, facilitating binding of the receptor binding region to the endosomal entry factor Niemann-Pick C1 protein (see below, 8.1. Entry) [94,95].

Besides its function in entry and budding, GP may also play a role in immune evasion. The IFN-inducible antiviral protein tetherin was shown to block the release of VP40-induced virus-like MARV and EBOV particles, suggesting that tetherin might act as a restriction factor for filovirus release [102,103]. However, co-expression of GP was sufficient to counteract the antiviral activity of tetherin by a yet unknown mechanism [104,105]. It is possible that GP not only subverts innate immune responses but also suppresses the adaptive immune response. Filoviral GP_2_ subunits, including MARV GP_2_, contain a domain resembling an immunosuppressive motif found in retroviral envelope proteins [106]. A 17-mer peptide corresponding to the putative immunosuppressive domain of MARV GP was shown to induce lymphocyte death and suppression of cytokine responses [107]. It is not yet known if this motif plays a role in the induction of lymphocyte apoptosis observed in MARV infection. Finally, it has been suggested that shedding of the ectodomain of membrane-bound EBOV GP by tumor necrosis factor α-converting enzyme (TACE) may play a role in blocking the activity of neutralizing antibodies during infection [108]. It has been reported for MARV that considerable amounts of GP shed from infected cells, although it is not clear if MARV GP is a target for TACE cleavage [108,109].

#### 7.3.2. Viral Protein 40 (VP40)

The matrix protein VP40 is encoded by the third MARV gene and is the counterpart of the M proteins of other NNS RNA viruses. VP40 plays a major role in the formation of virions by redistributing nucleocapsids from the perinuclear region to the plasma membrane, recruiting GP to the sites of budding, and mediating particle release [110,111,112]. Overexpression of VP40 led to reduced reporter gene expression of MARV minigenomes, suggesting a regulatory role of VP40 in transcription and/or replication [113].

As a peripheral membrane protein, VP40 coats the inner side of the virion’s membrane (Figure 5) [114]. Cryo-EM tomography studies suggest that VP40 associates with the nucleocapsid through flexible interactions [60]. It can be easily removed from the nucleocapsid by salt dissociation, indicating that it is only loosely connected to the nucleocapsid [115]. After synthesis in the cytoplasm of the infected cell, VP40 associates rapidly with cellular membranes and accumulates in membranous structures of the late endosomal compartment, the multivesicular bodies. A minor portion of VP40 is also found in association with viral nucleocapsids and in inclusions. Additionally, VP40 appears in patches beneath the plasma membrane where it is transported via the retrograde late endosomal pathway [62,114,116]. Similar to EBOV VP40, MARV VP40 is the major factor in particle formation and budding. Expression of VP40 in the absence of other viral proteins leads to the formation and release of filamentous virus-like particles (VLPs) resembling authentic virions. This process is enhanced in the presence of GP [113,117,118,119]. The role of VP40 during budding is described in more detail below (see 8.3. Budding).

Compared to EBOV VP40, little is known about the structure of MARV VP40. The N-terminal domain of MARV VP40 folds into ring-like structures, which have the tendency to polymerize into rod-like structures. While EBOV VP40 has been shown to form hexamers and octamers, the stoichiometry of MARV VP40 oligomers is not known [120]. MARV VP40 is phosphorylated at several tyrosine residues located in the N-terminal region of the protein. A non-phosphorylatable mutant of VP40 is impaired in its ability to recruit nucleocapsids to the sites of budding, but is still able to efficiently induce particle release [112]. VP40 also possesses a PPPY late domain motif in its amino terminus which is important for its interaction with components of the Endosomal Sorting Complex Required for Transport (ESCRT) machinery in order to mediate budding, including Tumor susceptibility gene 101 (Tsg101) and the membrane-bound E3 ubiquitin ligase Nedd4.1 [119,121,122]. Besides the PPPY motif, other motifs and single amino acids have been found to be important for particle release [123,124].

**Figure 6:**
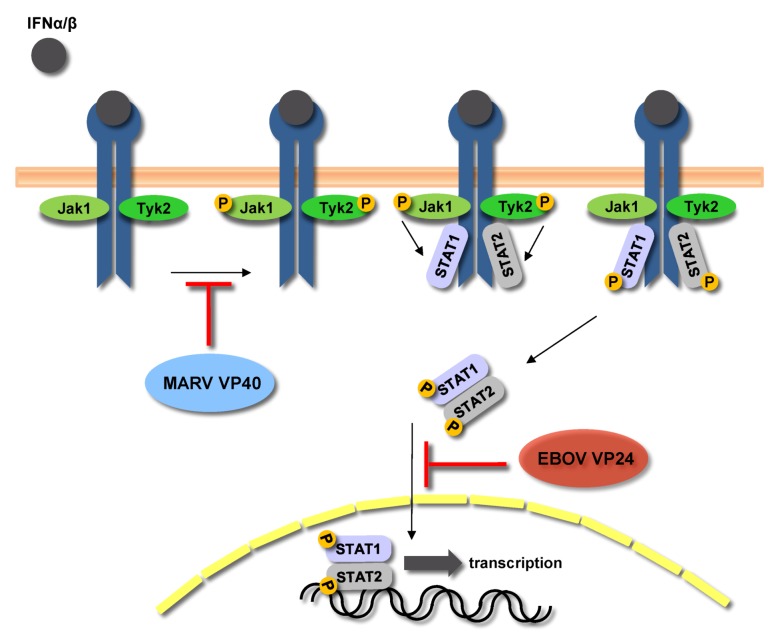
**Inhibition of JAK-STAT signaling by filoviruses. **MARV VP40 inhibits phosphorylation of Janus kinases and STAT proteins in response to Type I and II IFNs and IL6, preventing downstream signaling. Phosphorylation of STAT proteins is not inhibited by Ebola virus (EBOV). EBOV VP24 interacts with STAT1 and members of the nuclear importin family and prevents nuclear translocation of phosphorylated STAT1.

Besides its role as a classical matrix protein, MARV VP40 also acts as a virulence factor by counteracting the innate immune response and determining the host tropism for MARV [125,126]. MARV VP40 blocks the phosphorylation of Janus kinases, which play an important role in multiple signaling pathways by phosphorylating and activating STAT proteins (Figure 6). When MARV-infected cells were treated with various stimuli, including IFNα, IFNγ, and IL6, it was shown that the STAT proteins were neither phosphorylated nor translocated into the nucleus [126,127]. It was then shown that in MARV-infected cells treated with exogenous stimuli, Janus kinases were also not phosphorylated and VP40 was identified as the viral protein inhibiting IFN signaling. It is believed that Jak1 is the target for VP40, however, the mechanism of VP40-induced inhibition is not completely understood [126]. Intriguingly, EBOV is also able to block IFN signaling by employing a completely different mechanism. EBOV VP24 blocks the nuclear translocation of phosphorylated STAT proteins by binding to STAT1 and importins involved in the nuclear transport of specific STAT proteins (Figure 6) ([128], reviewed in [129]).

When MARV was adapted to non- or less permissive animals, such as mouse and guinea pig, the adapted viruses showed mutations in VP40. Two of the amino acid changes in the mouse-adapted MARV VP40 have been shown to be essential for the inhibition of IFN signaling in mouse cells, underlining the importance of IFN suppression for the virulence and host specificity of MARV [125,130,131].

#### 7.3.3. Viral protein 24 (VP24)

The protein product of the sixth gene, VP24, is unique to the filovirus family. VP24 is generally addressed as a second, minor matrix protein. However, cryo-electron tomography analysis of viral particles showed that VP24 is located in close proximity to the nucleocapsid proteins, suggesting that it might be part of the nucleocapsid complex [60]. VP24 can easily be released from virion-associated nucleocapsids by treatment with increasing salt concentrations, indicating that it is only loosely connected to the nucleocapsid [115]. Intracellular localization studies of VP24 showed that a minor part of the protein (approx. 10%) is weakly bound to cellular membranes, including filopodia enriched with VP40. VP24 is also distributed diffusely in the cytoplasm, relocalizes to nucleocapsid-containing inclusions, and is found in association with free nucleocapsids. Co-expression of NP and VP24 is sufficient to direct VP24 to NP inclusions in the cytoplasm [132]. Functional studies on MARV VP24 suggest that the protein is important for the release of viral particles in the context of infection, although it influences neither the morphology of VP40-derived VLPs nor the efficiency of VLP release. In addition, RNAi-mediated knockdown of VP24 in MARV-infected cells had no impact on viral genome replication, indicating that VP24 is involved in a step after replication and before budding [132]. According to the model that has been proposed based on these data, VP24 is involved in the maturation of transport-competent nucleocapsids and/or mediates the interaction between nucleocapsids and budding sites at the plasma membrane [132]. There is also evidence that MARV VP24 affects transcription and replication in a transcription and replication competent VLP system [113].

Structural information about MARV VP24 is very limited. It has been shown that it forms oligomers, preferentially tetramers [132]. Structure prediction studies have proposed an ancestral link between VP24 and the Armadillo repeat family [133].

#### 7.3.4. The Nucleocapsid Proteins NP, VP35, VP30 and L

The MARV nucleocapsid complex consists of the genomic RNA and four tightly associated proteins, NP, VP35, VP30, and L (Figure 5). Encapsidation of the viral RNA by the nucleocapsid proteins protects it from both RNase degradation and detection by cellular pattern recognition receptors. Similar to the genomic RNA, the antigenomic RNA, a replicative intermediate, is also encapsidated by the nucleocapsid proteins (see below, 8.2. Transcription and Replication). In contrast, the viral mRNAs are not encapsidated [134]. The nucleocapsid rather than naked RNA serves as the template for viral transcription and replication. In a MARV minigenome system, NP, VP35, and L are essential for transcription and replication [113,134]. The role of VP30 in MARV transcription and replication is not well understood and the steps in genome amplification that require, or do not require, VP30 are not yet defined.

#### 7.3.5. Nucleoprotein (NP)

The nucleoprotein NP enwraps the genomic and antigenomic RNAs. Replication and transcription activity in a MARV minigenome system depends on the presence of NP [134]. When NP is expressed in the absence of other nucleocapsid proteins, it self-assembles into highly organized helical tubular structures that resemble the nucleocapsids in infected cells, indicating that it is the driving force for nucleocapsid formation [60,135,136]. Recently, it has been shown that the conserved 390 N-terminal residues of MARV NP are sufficient to form the helical structure of the nucleocapsid core [60]. Indeed, NP serves as a viral hub protein. It forms interactions with most of the other viral proteins, leading to the subcellular redistribution of these proteins. The strong binding of NP to the nucleocapsid proteins VP35 and VP30 redirects both proteins into NP-derived inclusions [115]. A bipartite coiled-coil motif in the central part of NP has been shown to play an important role for self-assembly and NP-VP35 interaction [137]. As mentioned above, there is also a weak interaction between NP and VP24, leading to the partial relocalization of VP24 into NP-containing inclusions [60,132]. In addition, NP interacts with VP40, which is important for the transport of newly synthesized nucleocapsids to the plasma membrane [110,111,138,139]. Interestingly, NP contains a C-terminal late domain motif, PSAP, which has been shown to be required for budding. NP recruits Tsg101, a component of the ESCRT I complex, through its late domain motif, leading to enhanced VP40-induced budding [111].

NP is heavily phosphorylated at serine and threonine residues clustered in seven regions in the C-terminal part of the protein. Only the phosphorylated form of NP is incorporated into virions [140,141]. Recent studies suggest that the phosphorylation level in Region II modulates transcription and/or replication activity [142].

#### 7.3.6. Viral Protein 35 (VP35)

VP35 is a polymerase cofactor and essential for transcription and replication. Together with the catalytic subunit L, VP35 forms the RNA-dependent RNA polymerase complex [134,143]. VP35 is tightly associated with NP and serves as a bridging protein between the nucleocapsid complex and L. Without VP35, L is not associated with the nucleocapsids which serve as the templates for viral transcription and replication [115,134]. VP35 forms homo-oligomers mediated by a coiled-coil motif located in the N-terminal part of the protein. Homo-oligomerization of VP35 is essential for its interaction with L but not needed for redistribution of VP35 into NP-derived inclusions [144]. VP35 shares many features with the phospho (P) proteins of other NNS RNA viruses, including its position as the second gene in the viral genome and its role in transcription and replication. However, in contrast to the P proteins, VP35 is either not or only very weakly phosphorylated [145].

Besides its function in transcription and replication, MARV VP35 acts as an IFN antagonist. While the impact of EBOV VP35 on the host’s antiviral response has been intensively investigated (reviewed in [129]), much less information is available about similar functions of MARV VP35. When we tested MARV VP35 for its ability to block IFN induction in a reporter gene assay, it blocked reporter gene expression as efficiently as EBOV VP35 (unpublished data). In addition, Bosio and colleagues [146] reported that expression of MARV VP35 in the absence of other viral proteins was sufficient to completely block the induction of IFNα in stimulated human dendritic cells. Besides its ability to inhibit the induction of Type I IFN, EBOV VP35 has been shown to block the activation of the antiviral protein PKR and to interfere with RNA silencing pathways. Importantly, EBOV VP35 is a dsRNA binding protein. The C-terminus of EBOV VP35 contains a domain with patches of basic amino acids which is important for dsRNA binding and the protein’s inhibitory functions (for review see [129]). This C-terminal region, the so-called IFN inhibitory domain, is conserved in MARV VP35 [147], suggesting that MARV VP35 possesses similar inhibitory functions.

#### 7.3.7. Viral Protein 30 (VP30)

MARV and EBOV VP30 proteins show many structural similarities. Both MARV and EBOV VP30 proteins are tightly associated with the nucleocapsid via their binding to NP (Figure 5) [115,148]. Both are highly phosphorylated at N-terminally located serine and threonine residues, and phosphorylation is crucial for their interaction with NP [148,149]. Both contain an unusual C3H1 Zn binding domain, which is essential for the function of EBOV VP30 as transcription initiation factor, but whose functional relevance for MARV VP30 is not known [150]. It has also been shown that EBOV VP30 forms hexamers [151,152], binds single-stranded RNA [153], and interacts with L [154]. However, to date, similar data for MARV VP30 are not available.

The role of MARV VP30 in viral transcription and replication is not well understood. In contrast to EBOV VP30, which plays an important role in regulating transcription initiation [68,143,155,156,157], MARV VP30 is not essential for transcription or replication activity in a MARV minigenome system [113,134]. Nevertheless, it seems to play an important role in viral amplification, since rescue of a full-length MARV clone is only successful in the presence of VP30 [158]. In addition, down-regulation of VP30 by RNA interference in MARV-infected cells led to the reduction of both viral protein synthesis and virion production [159]. Among the NNS RNA viruses, only the members of the subfamily *Pneumovirinae* possess a protein similar to VP30, M2-1, which functions as a transcription processivity factor [160].

#### 7.3.8. Large Protein (L)

The major component of the MARV polymerase complex, L, has an estimated molecular weight of 267 kD [67]. It is essential for transcription and replication and together with VP35 forms the RNA-dependent RNA polymerase complex (see above, 7.3. Viral Proteins, VP35). L contains the enzymatic functions of the polymerase. The binding site for VP35 has been mapped to the N-terminal 530 amino acid residues of L [115,134]. The L proteins of the NNS RNA viruses are highly conserved multifunctional proteins, which are organized in functional domains [161]. Based on this conservation with other NNS RNA polymerases, MARV L is believed to carry out RNA synthesis, capping, and polyadenylation of viral mRNAs although these functions have not been shown experimentally.

## 8. Replication Cycle

To date most of the studies characterizing the MARV replication cycle have utilized recombinant systems, allowing for these experiments to be performed in a biosafety level 2 (BSL-2) context, unfettered by the restrictions of a BSL-4 setting. Surrogate systems mimicking specific steps in the MARV replication cycle include MARV GP-pseudotyped retroviruses or recombinant vesiculoviruses expressing GP to study entry, VLPs to study budding, and minigenome systems to study replication and transcription. While such experiments allow for the more facile examination of the MARV replication cycle, the findings must be recapitulated with infectious MARV since all surrogate systems lack elements of the infectious virus such as the distinct morphological features and virion protein composition of MARV.

### 8.1. Entry

**Figure 7:**
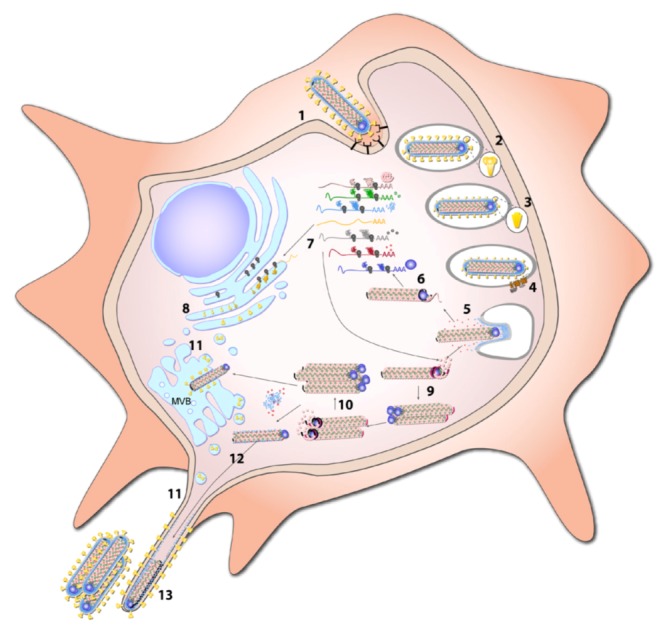
**Replication cycle. **MARV initially attaches to target cells via interaction with cell surface molecules (1). Following endocytosis (2), GP_1 _is cleaved by endosomal proteases (3) facilitating binding to NPC1, the entry receptor (4). Fusion is mediated in a pH-dependent manner by GP_2_. Following release of viral nucleocapsid into the cytosol (5), transcription of the viral genome takes place (6). mRNA is subsequently translated by the host cell machinery (7). Synthesis of GP takes place at the ER and undergoes multiple posttranslational modifications on its way through the classical secretory pathway (8). Positive sense antigenomes are synthesized from the incoming viral genomes (9). These intermediate products then serve as templates to replicate new negative sense genomes (10). After cleavage in the Golgi, GP is transported to multivesicular bodies (MVB) and to the cell membrane where budding takes place (11). Nucleocapsids and VP24 are also recruited to sites of viral budding (12), which is driven by VP40 (13).

Marburg virus entry consists of three distinct phases: cellular attachment, endocytosis, and fusion (Figure 7). Based on the sequence similarity between EBOV and MARV GPs many investigators have presumed identical functions and characteristics between the filovirus glycoproteins. This is presumptuous given the differences in glycosylation and sialic acid linkages [85] and dependence upon endosomal proteases (see below, 8.1.2. Endocytosis). Despite the existence of a number of detailed studies and structural analyses of EBOV GP [162,163,164,165], relatively few mechanistic studies of MARV GP have been performed [101], although one recent post-fusion structure of MARV GP_2_ has been reported [97]. The structure of MARV GP_2_ is nearly identical to that of EBOV GP_2_, indicating that the mechanisms of fusion between the two viruses is likely conserved [97]. 

#### 8.1.1. Attachment

MARV GP mediates both cell attachment and fusion of the virus. There is convincing evidence that initial virus attachment at the cell surface can occur via the binding of GP carbohydrates to various cellular C-type lectins, including the hepatocyte-specific ASGP-R [87], DC-SIGN and DC-SIGNR (also known as L-SIGN) [89,92,166], hMGL [91,92], and LSECtin [166,167]. Other cell surface proteins have also been implicated in facilitating MARV entry including the TAM receptor protein kinases Ax1, Dtk, and Mer [90], and TIM-1 [93]. However, although these proteins may play a role in attachment or entry of certain cell types, the ability of MARV to infect cells lacking these receptors [92,93,168] indicates that there might be redundancy in cellular molecules required for MARV attachment to cells.

A number of key residues of EBOV GP that are involved in virion incorporation and GP-mediated entry have been identified [163] and found to play a similar role in MARV GP [101], indicating that the viruses might utilize similar mechanisms to enter the cell. 

#### 8.1.2. Endocytosis

Following attachment, Marburg virions undergo endocytosis mediated through a mechanism that currently remains undetermined. (Figure 7) [16,62] Initial studies investigating caveolin-mediated endocytosis showed that depletion of host cell cholesterol reduced viral infectivity but presented no direct evidence of caveolae involvement [169]. In addition, studies examining the role of caveolae in EBOV endocytosis are conflicting [169,170]. A major role for clathrin in MARV entry has also been proposed based upon the ability of chlorpromazine (an inhibitor of both clathrin-mediated endocytosis and macropinocytosis) as well as RNAi-knockdown of clathrin heavy chain to inhibit MARV GP-pseudotyped HIV-1 entry [171]. A caveat to these analyses of MARV endocytosis is that they were performed only in the context of MARV GP-pseudotyped retroviruses which lack the characteristic filamentous morphology and size of Marburg virions.

While other reports have verified that cholesterol is important for live MARV particle uptake [172], canonical caveolae- and clathrin-mediated mechanisms are unlikely to be the primary mechanism of MARV entry due to steric issues. The typical MARV particle size (average 790 nm) is much larger than canonical caveolae (50–100 nm) or clathrin-coated pits (up to 200 nm) whereas pseudotyped murine leukemia virus (MLV) (100 × 100 nm) and vesicular stomatitis virus (VSV) (70 × 180 nm) are not [173]. These findings indicate that involvement of the caveolae- and clathrin-mediated endocytic pathways for virus entry may therefore be the result of the artificial nature of the pseudotyped virions and highlights the need to confirm such experiments with live MARV.

Macropinocytosis has been identified as a major entry pathway of EBOV by research using the morphologically more relevant VLPs and live EBOV [174,175,176,177]. Although none of these analyses examined the role of this pathway during MARV entry, it remains an intriguing possibility given the cholesterol-dependence and large size of macropinocytotic vesicles (up to 3–5 µm) [178].

Another important process in MARV entry is believed to occur while virions are being trafficked within endocytic vesicles; the proteolytic cleavage of GP_1_. Endosomal cleavage of GP has been shown to be critical for the efficient entry of MARV [179,180]. The current model for MARV entry involves the cleavage of GP_1_ by host endosomal cysteine proteases. This removal of a large portion of GP_1_ (including the mucin-like domain) is believed to expose the putative receptor-binding domain based on studies conducted with EBOV GP [181,182].

Studies examining the roles of endosomal proteases on the entry of MARV and EBOV have produced mixed results. Experiments analyzing recombinant VSV expressing EBOV GP indicate a primary role for Cathepsin B (CatB) and minor role for Cathepsin L (CatL) [181]. Entry of recombinant VSV particles containing MARV GP was inhibited when cells were treated with an inhibitor of both CatB and CatL [92]. These reports are confounded by a report conducted with infectious Marburg and Ebola viruses in which CatB and CatL inhibitors greatly reduced EBOV infection but showed mixed results with MARV [172]. Yet two other, more recent analyses determined that CatB was not required for MARV entry (although over-expression did enhance infectivity) and that CatL was required for entry into mouse embryonic fibroblasts but not Vero cells, 293T cells, or human macrophages [179,180]. These data as well as the ability of other proteases to greatly diminish MARV infectivity [179,180], indicate that although CatB and CatL likely play a role in cleavage and activation of GP_1_ in certain cell types, other endosomal proteases may also be able to facilitate GP_1_ activation via cleavage.

#### 8.1.3. Fusion

Recently, two independent studies elegantly showed the requirement of the endosomal cholesterol transporter Niemann-Pick C1 (NPC1) for the entry of both MARV GP-pseudotyped viruses (VSV and MLV) as well as infectious MARV [94,95]. It was also shown that NPC1 catalytic activity is not required for EBOV infection indicating that specific binding to NPC1 rather than its role in cholesterol transport is required, although this was not tested for MARV [95]. In one of the studies identifying NPC1 as the MARV entry receptor, it was also determined that members of the homotypic fusion and vacuole protein-sorting (HOPS) complex were important for EBOV entry, although they appeared to play a less important role in MARV entry [94].

The current model of EBOV and MARV fusion is that GP_1_ cleavage by endosomal proteases removes heavily glycosylated domains, exposing the receptor binding domain on GP_1_ and enabling binding to NPC1 [95]. The membrane-bound fusogenic GP_2_ undergoes a low pH-dependent rearrangement to an extended conformation resulting in the fusion of virion and endo-lysosomal membranes [96]. In support of the pH-dependence of GP-mediated fusion, pre-treatment of cells with ammonium chloride prevented entry of a MARV GP-pseudotyped virus [183]. A recent report with live MARV showed that ammonium chloride inhibited entry and replication, but that Bafilomycin A_1_, which specifically inhibits vacuolar-type H(+) ATPase and prevents re-acidification of vesicles of the central vacuolar system, surprisingly had no effect [172]. Following viral fusion with the endosomal membrane, the nucleocapsid is released into the cytoplasm (Figure 7).

### 8.2. Transcription and Replication

After the nucleocapsid is released into the cytoplasm of the infected cell, transcription and replication of the viral RNA genome takes place (Figure 7). The first morphological sign of viral replication observed by EM analysis is the appearance of granular material containing RNA and viral proteins in the cytoplasm of the infected cells at 12 h post infection. Later on, tubular structures can be detected in the granular material representing the newly synthesized nucleocapsids embedded in the viral inclusions [63]. While experimental data on the sites of MARV replication and transcription are not available, recent studies on EBOV have shown that viral replication takes place in the inclusions, while transcription was observed prior to inclusion formation [184].

The encapsidated negative-sense RNA genome is transcribed resulting in seven monocistronic mRNAs by the viral polymerase. They are co-transcriptionally capped and polyadenylated and subsequently translated by the cellular machinery (Figure 7). The genomic RNA also serves as the template for the production of positive-sense antigenomes, which are complementary copies of the genomes. The antigenomes are encapsidated by the nucleocapsid proteins and are in turn used as templates for genome synthesis (Figure 7) (for review see [68]). As mentioned above, NP, VP35, L, and probably VP30 are needed for viral transcription and replication. Analogous to EBOV, it is conceivable that VP40 and VP24 inhibit transcription and replication [113,185,186]. It is hypothesized that negative regulators of replication convert the active polymerase complex into an inactive state, resulting in mature and transport-competent nucleocapsids.

### 8.3. Budding

Following assembly, newly synthesized nucleocapsids are recruited to the sites of virus budding (Figure 7). Release of viral particles is mainly mediated by VP40 via recruitment of nucleocapsids from the inclusions to the plasma membrane, recruiting GP to the sites of budding, and inducing the formation and release of filamentous VLPs. VP40-induced budding is enhanced by NP, GP, and VP24 [98,111,132]. As is the case with many other viruses, MARV exploits the vesicular transport machinery of the infected cell for viral egress, including the COPII vesicular transport system and the ESCRT machinery. The COPII vesicular transport system is used by VP40 for its intracellular trafficking to the multivesicular bodies, where MARV budding takes place [118,187]. Cellular proteins that promote particle release and are linked to the ESCRT machinery include Tsg101, Vps4A/B, and Nedd4.1 [111,119,121,122]. MARV budding not only takes place at internal membranes but also at the plasma membrane [63,118,188]. In cell culture, MARV particles are preferentially released at filopodia, filamentous cellular protrusions [60,188,189]. Filopodia are used by cells to explore the extracellular environment, which includes neighboring cells, and it is believed that viral particles can bud directly into adjacent cells via filopodia-mediated cell-to-cell contact [188,190]. Budding at filopodia depends on actin and is not sensitive to the depolymerization of microtubules [188]. MARV budding was observed at the basolateral membrane of polarized epithelial cells and hepatocytes [109,191], whereas viral particles were predominantly released from the apical membrane of infected endothelial cells [64], suggesting that cell-type specific components determine the sites of virus release.

Electron tomography studies of MARV-infected cells led to the following model for MARV particle release: The budding process is initiated when intracellular nucleocapsids associate laterally with the plasma membrane. Starting from one end, the nucleocapsids are then subsequently wrapped by the plasma membrane until the viral particles protrude vertically from the cell surface. The release of infectious filamentous MARV from cultured cells peaked at 1–2 days post infection, when the cells were still intact. At 4 d post infection, when most of the cells were vesiculated, the released virions were round or bent and infectivity was decreased [189]. Determination of the nucleocapsid orientation at the sites of budding by 3-D reconstructions revealed that the pointed tip of the budding nucleocapsids is oriented towards the membrane, indicating that MARV budding is directional [60].

## 9. Pathogenesis

MARV infections usually occur by direct contact with infected body fluids or direct personal contact with infected animals or humans. The viruses enter the body through small skin lesions or mucosal membranes (reviewed in [47]). Cells of the mononuclear phagocyte system, including monocytes, macrophages and dendritic cells, are early target cells of MARV, as shown in different experimental animal models [14,192,193,194,195,196,197]. MARV replication was observed as early as 24 hours post infection in macrophages of infected guinea pigs [63], and infected monocytes have been found in cynomolgus macaques at 2 days post infection [193]. Monocytes and macrophages were also identified as early target cells in human patients [197]. This has been confirmed by cell culture experiments showing that primary human monocytes and macrophages are highly susceptible to MARV infection and produce infectious virus [198,199,200]. In addition, primary human monocyte-derived dendritic cells (mDCs) and endothelial cells support MARV replication [64,146,201].

Early sites of virus replication are the lymph nodes, liver, and spleen where the most severe necrotic lesions are observed [8,13,14,41,130,194,202].

These organs contain high numbers of monocytes and macrophages. Migration of infected monocytes and macrophages into surrounding tissues or transport of free virus via the lymph- or bloodstream is believed to facilitate the dissemination to multiple organs, resulting in a systemic infection [203,204]. Cell-free virus has been observed in the tissue and organs of infected animals, and high levels of virus have been detected in the blood [12,13,14,130,194,195,205,206]. Besides monocytes, macrophages, and dendritic cells, a wide range of cell types including hepatocytes, adrenal cortical and medullary cells and fibroblasts are permissive to MARV infection [12,14,192,193,194,195,197,201,207]. Endothelial cells are late target cells during MARV infection in multiple tissues. Whether or not replication of MARV in endothelial cells is associated with the observed vascular impairment during MVD is a matter of debate [64,194]. Only low numbers of infected endothelial cells are observed in NHP infection and therefore changes in the endothelium are likely caused by paracrine effects of cytokines [14].

In late stages of infection MARV particles can be isolated from nearly every organ [12,14,130,194,208]. Despite high viral load and necrotic lesions, only minor inflammation is observed in infected tissues and organs, indicating a dysregulated immune response [8,14,41,194]. Strong liver pathology is observed, including increased serum activity of liver enzymes. This might influence synthesis of clotting factors and contribute to the observed coagulation defects in MVD [12,41,130,194]. These factors together with systemic virus replication and associated pathology probably trigger the multiorgan failure associated with fatal cases.

Although lymphocytes are not susceptible to MARV infection [14,193,194,201], massive bystander lymphocyte apoptosis is a hallmark of MVD [8,14,130,194,195,201]. However, the molecular mechanisms for lymphocyte depletion and the role it may play in the pathogenesis of MVD are far from being understood.

Cytokine secretion may play a role in the induction of lymphocyte apoptosis, since MARV-infected cells secrete cytokines known to induce apoptosis, including TNFα [194,198,200,209]. Increased levels of TNFα have been observed in infected rhesus macaques [210] and mice [130], although no increase was observed in infected cynomolgus macaques [14]. Elevated TNFα levels may also play a role in the formation of endothelial gaps in the context of MARV infection [198,199]. In addition, increased survival of MARV-infected guinea pigs treated with anti-TNFα antibodies suggests that TNFα indeed plays an important role in MVD pathogenesis [209].

Increased serum levels of additional proinflammatory cytokines and chemokines have been observed in infected NHPs and in mice, but the reported data are not completely consistent [12,14,130,193,210]. Cytokine and chemokine secretion has also been observed in infected primary human monocytes and macrophages [200,211]. However, data about the cytokine levels in the serum of MARV-infected patients are not available, but high levels of cytokines have been observed in EBOV-infected patients [212,213,214,215].

Upregulation of the proinflammatory cytokines IL6 (mediator of fever and acute inflammatory response) and IL8 (chemoattraction of neutrophils and macrophages) is consistently found in infected NHPs, with macrophages and plasmacytoid dendritic cells (pDCs) serving as the main sources of IL6 secretion in the spleen [12,14,193]. Primary human monocytes and macrophages produce both IL6 and IL8 after infection [200]. Elevated levels of IL6 have also been detected in MARV-infected mice [130]. Increased levels of IL1β mRNA and secreted protein were detected in primary human cells [200,211], but contradictory data have been reported for the NHP model. One study reported elevated IL1β levels in final disease stages [12], whereas no change was observed in another study [14].

IFNα levels were elevated in infected NHPs and mice [130,193,210]. However, no change in IFNα levels was detected in another study of infected NHPs [14]. It is unclear whether or not the observed differences are due to different MARV variants being used for the studies.

Serum levels of several chemokines were also found to be elevated during MARV infection of NHPs and mice, including macrophage inflammatory proteins (MIP) and monocyte chemotactic protein 1 (MCP-1) [12,14,130].

The involvement of multiple cell types along with the possible role of non-infected cells in the secretion of cytokines further complicates the analysis of existing data. Primary human monocytes and macrophages are activated by MARV infection inducing the secretion of cytokines. Induction of cytokines has also been described using UV-inactivated MARV, suggesting that viral replication might not be needed for the observed cytokine increase [200]. In contrast, MARV-infected mDCs show no upregulation of activation markers, do not secrete cytokines, and fail to stimulate T cells [146]. mDCs treated with VLPs containing MARV VP40 and GP show functional mDC responses including cytokine secretion indicating that MARV replication is required to inhibit mDC activation [216]. However, infection of mDCs with MARV did not prevent LPS-induced TNFα production whereas dsRNA-dependent IFNα secretion was inhibited [146], suggesting differential regulation of cytokines by MARV. Interestingly, pDCs in the spleen were identified as the major source of secreted IFNα in MARV infected NHPs, but secretion most likely occurs from non-infected cells [193]. It has been shown for EBOV that pDCs are not productively infected due to impairment of viral entry [217]. These results suggest that secretion of cytokines by non-infected bystander cells might play an important role during MARV pathogenesis. Taken together, MARV infection induces both an increase in the production of proinflammatory cytokines and high levels of chemokines, but the molecular mechanisms causing these changes are not well understood.

## 10. Animal Models

To date four different animal models have been established for MARV infection: NHPs, mice, guinea pigs, and hamsters. The NHP model best reflects the symptoms and pathology observed in human cases (described in 5. Clinical Manifestations and reviewed in [46,48]) with uniform lethality in cynomolgus and rhesus macaques as well as African green monkeys [12,14,26,193,194,206,218,219,220]. The disease symptoms are generally the same for all types of NHPs. The animals develop febrile illness with high fever, anorexia, weight loss and unresponsiveness. Death is observed after 6–13 days and thrombocytopenia, lymphopenia, blood coagulation abnormalities and hemorrhages are observed. Squirrel monkeys have also been successfully infected with MARV, showing typical disease symptoms [206]. Recently, a small NHP model using marmosets has been developed recapitulating the features of human infections except for the typical maculopapular rash development that is observed in other NHPs and humans infected with MARV [221]. Rodents with an intact immune system do not develop disease after infection with MARV. MARV variants Musoke and Ci67 and RAVV variant Ravn were adapted to severe-combined immunodeficiency (scid) mice by serial passaging, reducing the time to death from 50–70 days to 7–10 days for all tested virus variants [195]. Further passaging of the scid mouse-adapted marburgviruses in immunocompetent mice was used to establish mouse models for RAVV Ravn and MARV Ci67 [130,131]. Successful adaptation by serial passaging was also used to generate lethal infection models for both guinea pig [218,222] and hamster [206,208]. Coagulation abnormalities, typical rash development, and hemorrhagic manifestations (especially in mice) are not as pronounced as in the NHP model [130]; (reviewed in [223]). Neuropathogenicity, recapitulating the CNS involvement described during the first human MVD outbreak in Germany [41,224,225], has only been observed in the hamster model [208]. It is not clear if CNS pathology is developed in other animal models as no brain pathology has been observed in mice [195] and cynomolgus macaques [14]. Nevertheless, virus has been isolated from brain from MARV-infected marmosets, showing micro-hemorrhages [221].

Sequence comparison of the rodent-adapted viruses to the human MARV isolates revealed several mutations. Sixty-one nucleotide changes in the mouse-adapted RAVV Ravn variant were detected in the ORFs of NP, VP35, VP40, and VP30 (14 amino acid changes in total) or untranslated regions (VP35, VP40, GP, VP30) [130]. In a second study analyzing genome changes during mouse adaptation, 75 nucleotide changes during adaptation of RAVV Ravn and 33 changes for MARV Ci67 were described, with most amino acid changes occurring in VP40 [131]. During guinea pig adaptation, only 11 nucleotide changes were observed, resulting in four amino acid changes. One of these changes was located in VP40 and the other mutations were detected in the viral polymerase L [222]. The only amino acid exchange detected in both the mouse- and the guinea pig-adapted MARV is amino acid 184 in VP40 (Asp to Asn). This was also the first mutation detected during mouse adaptation for both RAVV Ravn and MARV Ci67, as analyzed by sequencing of serial passages [131]. This is of particular interest because VP40 has been shown to function as an inhibitor of IFN signaling (see above, 7.3. Viral Proteins, VP40) [125,126]. Both IFN receptor- and STAT1-deficient mice develop disease using non-adapted MARV, highlighting the importance of the IFN pathway for the control of MVD [226,227,228].

## 11. Diagnosis

Virological, serological, and molecular diagnostic methods for the detection for MARV are available, including virus isolation, ELISA, RT-PCR, EM, and immunohistochemistry (summarized in [16,46]). During outbreak settings, mobile laboratories commonly use PCR and/or ELISA analysis for rapid screening. Sensitive ELISA assays have been developed for detection of viral antigen or virus-specific antibodies using overexpressed MARV NP or GP [229,230,231,232]. Detection of filoviruses by PCR is the only assay currently available to distinguish between different virus variants for a variety of tissue and fluid specimens. The use of a combination of virus-specific primer sets for conventional RT-PCR makes the detection of all know filoviruses in a single assay possible [233]. For more sensitive and quantitative detection, real-time RT-PCR-based assays have been developed for the detection of MARV [10,234,235,236,237,238]. Feasibility of real-time RT-PCR in the field was successfully proven during the MARV outbreak in Uíge, Angola using an improved field laboratory-adapted RNA isolation protocol [239]. A network of European BSL-4 facilities in collaboration with a company (QIAGEN) developed the first commercial prototype of a real-time-RT-PCR assay for the detection of filoviruses [240]. A recently developed assay, RT loop-mediated isothermal amplification (LAMP), has the potential to significantly improve field diagnosis of MARV infections, by eliminating the need of PCR machines [241].

## 12. Vaccine Development

Initial approaches using inactivated virus to develop a vaccine against MARV were unsuccessful or had contradictory results [16]. In addition, successful protection of rodents did not always translate into protection of NHPs. For example, inactivated MARV protects guinea pigs from lethal MARV challenge but only 50% of challenged NHPs survived [210,242,243,244]. A panel of different approaches has been used in order to develop successful vaccines for MARV (reviewed in [245,246]).

Recombinant GP expressed from insect cells or a DNA vaccine based on GP only partially protected guinea pigs, but use of a combination of both vaccines resulted in 100% survival of guinea pigs [243]. In another study, complete protection of guinea pigs using a different GP DNA vaccine was reported, but only four of six vaccinated NHPs survived the challenge with MARV, showing incomplete protection [247]. Increased doses of a codon-optimized DNA vaccine resulted in 100% survival of NHPs, although some animals developed symptoms before recovering. In comparison to other vaccine candidates, a poor induction of virus-specific antibodies was observed using a DNA vaccine [248]. A codon-optimized DNA vaccine elicited a strong antibody response and resulted in complete protection of mice with no clinical symptoms observed [249]. Vaccine candidates based on the Venezuelan equine encephalitis virus (VEEV) replicon system expressing either MARV GP along with NP or GP alone completely protected guinea pigs and NHPs [250].

A vaccine based on VLPs represents an additional candidate for protection against MARV [251]. Complete protection of guinea pigs has been demonstrated with a VLP-based vaccine containing MARV GP, with induction of virus-specific antibodies. Protection with this vaccine relied on a functional CD4+ T cell response, whereas depletion of CD8+ T cells did not ablate the protective response [244]. VLPs containing MARV Musoke GP provided cross-protection in animals challenged with MARV Ci67 or RAVV Ravn in both guinea pigs and NHPs [252,253].

Another approach to MARV vaccines is the use of viral vectors expressing MARV GP. To date, two different systems have been established based on replication-defective adenoviral vectors or recombinant VSV expressing MARV GP. The adenovirus-based vaccine successfully protects guinea pigs and NHPs, and provides cross-protection. High levels of cross-reactive MARV-specific IgG and T cell responses are induced, indicating an induction of an immune response [248,254]. Preexisting immunity against the adenovirus strain Ad5 might pose a problem for its successful use in humans (reviewed in [245]).

The VSV-based vaccine completely protects NHPs and additionally has proven successful in post-exposure treatment (reviewed in [255]). A single immunization with recombinant VSV expressing MARV Musoke GP resulted in 100% protection of cynomolgus macaques challenged by intramuscular injection or aerosol exposure and protected against RAVV Ravn and MARV Angola [13,256,257]. Although MARV-specific IgG were produced, only low levels of neutralizing antibodies were detected [13,257]. Surprisingly, T cell-mediated responses were not observed in NHPs vaccinated with recombinant VSV expressing MARV GP [13,256]. Safety is a concern for this vaccine, especially for immunocompromised individuals, as it is a replication-competent VSV vector. However, in all VSV-based filovirus vaccine studies VSV viremia was observed only shortly after immunization. Additionally, the VSV-based filovirus GP vaccine was well tolerated and protective in immunocompromised mice and NHPs and lacked neurovirulence in NHPs [258,259,260] (reviewed in [255]).

Cross-protection has not been observed in animals vaccinated with MARV-based vaccines and subsequently challenged with EBOV, while combined MARV and EBOV vaccines have been successful in protection against both viruses [252,261,262].

## 13. Treatment

To date no approved treatment is available for MARV infection. Supportive care (fluids, anti-microbials, blood transfusions) has been the primary treatment of patients during MVD outbreaks. In the guinea pig model various treatments had some success as reflected by prolonged survival or increased survival rates. Applied treatments included cytokine inhibition, IFN treatment, or antibody transfer. The tested treatments were unsuccessful in the NHP model (reviewed in [16,47]).

A third of EBOV-infected NHPs survived, however, following treatment with recombinant nematode coagulant protein 2, while only one of six MARV-infected animals survived [12,263]. Treatment using antisense technology to block viral protein expression using phosphorodiamidate morpholino oligomers (PMO) beginning 30 to 60 minutes after MARV infection completely protected NHPs [264]. Additionally, a small molecule inhibitor showed complete protection of MARV-infected mice when administered 24h after infection but has not been tested in NHPs [265].

The VSV-based vaccine expressing MARV GP has also been demonstrated to be effective as a post-exposure treatment. A hundred percent survival of NHPs was observed when the vaccine was administered 20 to 30 minutes after MARV infection [266]. Delaying the time before treatment results in incomplete protection, although five of six animals or two of six animals still survived when given the treatment 1 or 2 days after MARV exposure, respectively [205]. These post-exposure treatments may be useful to prevent disease after known exposure to MARV, such as a laboratory accident, but the effective time frame during an outbreak might be too short and alternatives are needed.
